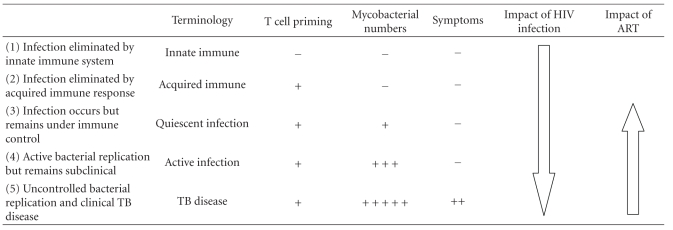# Changing Concepts of “Latent Tuberculosis Infection” in Patients Living with HIV Infection

**DOI:** 10.1155/2011/980594

**Published:** 2010-09-26

**Authors:** Stephen D. Lawn, Robin Wood, Robert J. Wilkinson

**Affiliations:** ^1^The Desmond Tutu HIV Centre, Institute for Infectious Disease and Molecular Medicine, Faculty of Health Sciences, University of Cape Town, Anzio Road, Observatory 7925, Cape Town, South Africa; ^2^Clinical Research Unit, Department of Infectious and Tropical Diseases, London School of Hygiene and Tropical Medicine, London WC1E 7HT, UK; ^3^Clinical Infectious Diseases Research Initiative, Institute of Infectious Disease and Molecular Medicine and Department of Medicine, Faculty of Health Sciences, University of Cape Town, Observatory 7925, South Africa; ^4^Division of Mycobacterial Research, MRC National Institute for Medical Research, Mill Hill, London NW7 1AA, UK; ^5^Division of Medicine, Imperial College London, London W2 1PG, UK

## Abstract

One third of the world's population is estimated to be infected with *Mycobacterium tuberculosis*, representing a huge reservoir of potential tuberculosis (TB) disease. Risk of progression to active TB is highest in those with HIV coinfection. However, the nature of the host-pathogen relationship in those with “latent TB infection” and how this is affected by HIV coinfection are poorly understood. The traditional paradigm that distinguishes latent infection from active TB as distinct compartmentalised states is overly simplistic. Instead the host-pathogen relationship in “latent TB infection” is likely to represent a spectrum of immune responses, mycobacterial metabolic activity, and bacillary numbers. We propose that the impact of HIV infection might better be conceptualised as a shift of the spectrum towards poor immune control, higher mycobacterial metabolic activity, and greater organism load, with subsequent increased risk of progression to active disease. Here we discuss the evidence for such a model and the implications for interventions to control the HIV-associated TB epidemic.

## 1. Introduction

Tuberculosis (TB) remains a major challenge to global public health in the 21st century [1]. In 2008, there were estimated 9.4 million incident cases and 11.1 million prevalent cases [2]. These resulted in 1.3 million TB deaths in HIV-negative individuals and additional 0.5 million deaths in HIV-infected individuals. This immense burden of disease is fuelled by very high rates of infection with *Mycobacterium tuberculosis, *estimated to occur in approximately one third of the world's population [3]. With current rates of progress, the millennium development goal (MDG) and associated World Health Organization (WHO) Stop TB targets for TB control will not be achieved by 2015 [4]. 

The failure to control TB in modern times stems, at least in part, from complacency towards TB control in the 1970s and 1980s and the subsequent devastating impact of the HIV-1 pandemic and may be further shaped by other important emerging epidemiological factors at a global level [1, 5]. Progress has also been hindered by the limited progress in developing more effective tools such as point-of-care diagnostics and treatments for active and latent disease, preventive vaccines, and laboratory assays of immune protection and cure. This lack of progress is, in turn, related to a poor understanding of the fundamental relationship between *M. tuberculosis *and the human host and especially the nature of what is referred to as “latent tuberculosis infection”.

Although *M. tuberculosis *was described as the aetiological agent of TB in 1882, fundamental insights into TB infection and disease have altered little for many decades. By analogy, the contrast between TB and HIV is stark. HIV is a relatively new pathogen discovered less than 30 years ago, and yet rapid advances in understanding pathogenesis were made quickly. HIV transmission is well understood and diagnosis is rapid, cheap, and reliable. There are quantitative tests of both HIV load and the impact on the host immune system, which have strong prognostic significance. Completely new treatments have been developed and have transformed prognosis.

A pivotal advance in addressing the HIV/AIDS epidemic was made in the mid-1990s when our understanding of the host-pathogen relationship completely altered. Prior to this time, HIV-infected patients were classified as having a clinically “latent” phase lasting many years between the seroconversion and the later symptomatic phase. It was generally assumed that the virus was similarly quiescent during this asymptomatic phase. However, advances in molecular techniques that permitted direct measurements of plasma HIV-1 load and CD4 lymphocyte counts and the advent of combination antiretroviral drug therapy resulted in a complete paradigm shift. “Clinically latent” HIV infection was discovered to be characterised by phenomenally high rates of virion replication and CD4 cell turnover [6, 7]. This observation has been fundamental to our approach to treatment and prevention.

Traditional views of “latent TB infection” are also being questioned. However, the laboratory tools with which to study this still lag considerably behind those of the HIV research field and obtaining samples from sites of disease is more challenging. In this paper we discuss the changing paradigm of infection and disease and draw on clinical and epidemiological observations to consider how this may be especially important to understanding the disease spectrum in those living with HIV infection. We further discuss the implications of this changing view to approaches for control of the HIV-associated TB epidemic. 

## 2. “Latent” Tuberculosis Infection

### 2.1. The Conventional Paradigm

The conventional view recognises TB infection and TB disease in human subjects as distinct binary states. Transitions between these states are described as resulting in one of three main clinical outcomes—“primary TB disease”, “latent TB infection” and “reactivation or post-primary TB disease.” In a minority of subjects (especially children), initial immune containment fails and infection progresses usually within 1-2 years to disease (primary TB). In the majority, however, immune responses control and limit the infection, such that individuals remain free from disease for prolonged periods but have evidence of infection defined by immunological sensitization to the organism. Evidence of this state of “latent TB infection” is therefore provided by positive tuberculin skin test (TST) responses or interferon-gamma release assays (IGRAs) incorporating species-specific mycobacterial proteins [8]. A small minority of such individuals will at some stage during their lifetime develop disease (“reactivation” or “post-primary TB”), in which loss of control of bacillary replication leads to development of TB disease.

### 2.2. Limitations of the Conventional Paradigm

The inadequacy of the existing compartmentalised view of TB infection and disease is apparent from the profusion of confusing terminologies used in the literature. Such terms include latent, active, inactive, subclinical, acute, chronic, persistent, dormant, recent, remote, quiescent, and incipient infection or disease. Collectively, these suggest that the host-pathogen relationship might better be considered as a spectrum.

A number of lines of evidence support the notion that the existing paradigm is an oversimplification (Table 1). The risk of TB among patients with “latent TB infection” changes considerably over time, depending strongly on how recently the infection was acquired [11]. This suggests that there is heterogeneity over time in the biology of “latent TB infection”. Evidence of such heterogeneity also comes from frequently observed discordance between the TST responses and IGRA results. These differences cannot simply be explained by lower specificity of the TST; a proportion of patients are IGRA positive but TST negative and others are IGRA negative but TST positive. Reversion from positive to negative appears to occur more commonly for IGRAs than TST responses [9]. This may relate to the fact that IGRAs tend to record the presence of *M. tuberculosis*-specific effector memory and terminally differentiated effector memory cells [12], and the persistence of these cells is thought to require continued antigen stimulation. In contrast, TST reactions also contain a proportion of cells of central memory phenotype [13], which may contribute to the development of positive TST reactions in the absence of long-term infection. 

The use of 6–12 months of isoniazid preventive therapy (IPT) is associated with a TB risk reduction of 60% (95% CI, 48–69) in HIV-uninfected individuals [14] and may lead to eventual reversion of TST status, suggesting possible eradication of infection [15]. Isoniazid acts by inhibiting mycobacterial cell wall synthesis and is therefore only active against actively replicating organisms. However, despite the fact that the number of organisms present is likely to be much lower than the numbers present in those with active disease, IPT is effective when given in courses lasting 6–12 months. This suggests that only a proportion of bacilli in patients with “latent TB infection” are replicating at any given time. A possible explanation is that “latent” *M. tuberculosis* bacilli cycle through a range of metabolic states over time, cumulatively rendering a large proportion of the pool of latent organisms susceptible to the drug during 6–12 months of preventive therapy. Thus, what has been referred to as “latent TB infection” may actually represent a dynamic balance between the organisms in a range of metabolic states and variations in immune control.

Data from TB prevalence surveys also indicate that a rigid binary distinction between “latent TB infection” and active symptomatic TB disease is oversimplistic. For very pragmatic reasons, TB control programmes in resource-limited settings have usually defined pulmonary TB suspects as those who have been coughing for at least 2-3 weeks [16]. However, during TB prevalence surveys, culture-proven TB is diagnosed in a proportion of patients who have no symptoms of disease at all [17–19]. For example, in a large survey of bacteriologically confirmed pulmonary TB in Vietnam, cough for more than 2 weeks was reported by just 55% of patients, cough of any duration was reported by 67%, and any symptom suggestive of TB was reported by 74% of patients [19]. Thus, approximately one in four patients was symptom-free at the time of the survey. 

If left untreated, patients with asymptomatic active TB may progress to develop symptomatic disease over time as observed among HIV-infected patients in Zimbabwe [20]. Thus, asymptomatic active TB may be an intermediate disease state between “latent TB infection” and overt symptomatic disease. This concept is supported by novel serological assays, which also provide evidence for a spectrum of disease stages, with patients with clinical disease and subclinical TB producing antibodies to a range of different stage-specific mycobacterial proteins [21–23]. 

In those with TB disease, clinical manifestations are also extremely diverse depending on anatomical site of disease, bacillary numbers (paucibacillary and multibacillary), and the host inflammatory response. Even within the same individual, diverse lesions ranging from sterility to multibacillary disease have been observed in a primate model of TB, suggesting that both “active” and “latent” lesions may coexist in the same individual [24].

## 3. Proposed Alternative Paradigm of “Latent” Tuberculosis Infection

**Figure 1:**
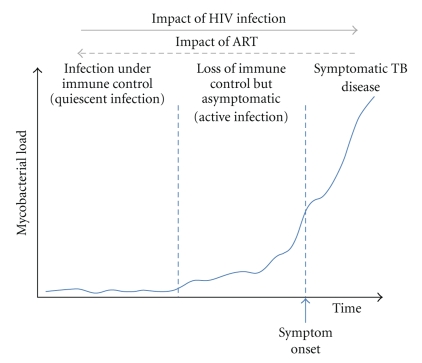
A conceptual stylised diagram showing rising total bacillary load of *Mycobacterium tuberculosis* over time in an infected patient. The patient initially retains good immune control and low bacillary numbers (quiescent infection). Subsequent loss of immune control is associated with rising bacillary numbers (active infection) and eventual development of symptoms (TB disease). The risk of transition from latent infection to active infection and disease is increased markedly by HIV coinfection whereas antiretroviral therapy would tend to enhance immune control.

There is now growing support within the field that the nature of the relationship between *M. tuberculosis* and the human host represents a spectrum of immune responses, mycobacterial metabolic activity, and organism load. In two viewpoints, Young, Barry, and colleagues have divided this spectrum into five different states that represent a dynamic spectrum ranging from immunity to TB disease (Table 2) [9, 10]. Following exposure to *M. tuberculosis*, infection may either be eliminated by the innate immune response (without the need for T cell priming) or eliminated following development of an acquired immune response. Such states may be referred to as “innate immune” and “acquired immune”, respectively (Table 2). Among those who are unable to prevent or eliminate infection following exposure, a majority establish and maintain immune control. Such individuals typically have evidence of T cell priming and maintain low bacillary numbers (quiescent infection). A subsequent shift in the host-pathogen response, however, may permit active mycobacterial replication, leading to subclinical active infection (Figure 1). The relationship between “quiescent” and “active” infection is likely to be a dynamic one over time with bidirectional shifts between the two. Loss of immune control and escalating mycobacterial load, however, may subsequently lead to the development of symptoms and overt clinical disease (Figure 1). 

## 4. Impact of HIV on the Proposed Host-Pathogen Relationship

### 4.1. Hypothesis

HIV infection is the most potent risk factor for the development of TB disease. The existing understanding of this is that HIV leads to an increased risk of rapidly progressive primary TB following exposure and also an increased risk of reactivation of “latent TB infection” to active disease. However, rather than increasing the risk of transition between compartmentalised disease states, we hypothesize that HIV coinfection has a fundamental impact on the spectrum of the host-pathogen relationship with a general shift towards poor immune control, high bacillary numbers, and subsequent development of active infection and symptomatic disease (Figure 1; Table 2). Recurrent exogenous re-exposure to *M. tuberculosis *in high TB prevalence settings is also very likely to play an important role, further increasing bacillary numbers and increasing the likelihood of progression to disease. In the remainder of this paper, we consider evidence for this hypothesis and the implications for disease control strategies.

### 4.2. Epidemiological Observations

Although HIV impairs some aspects of innate immune responses to *M. tuberculosis *[25], the impact of risk of acquisition of infection following exposure is unknown. Studies of intravenous drug abusers in New York early in the HIV epidemic did not find an increased risk [26] but the conclusion is undermined by the fact that assessment of the presence of infection was made using the tuberculin skin test, which has impaired sensitivity in those with HIV infection. Other data have shown very high rates of recurrent TB among HIV-infected individuals due to reinfection [27], possibly indicating high susceptibility to reinfection. However, this aspect of the host-pathogen relationship remains poorly characterised. 

Following acquisition of HIV-1 infection, the risk of TB in individuals with *M. tuberculosis* infection increases from an approximately 10% lifetime risk to over 10% each year [5]. The risk in those living with HIV depends on the degree of immunodeficiency, the prevailing socioeconomic conditions, and the TB infection pressure in the community. The rapidity with which risk increases following HIV seroconversion is striking, rising 2-3-fold within the first 2 years of infection even prior to substantial depletion of the blood CD4 cell count [28, 29]. In a primate of model of TB and simian immunodeficiency virus (SIV) coinfection, risk of reactivation TB was associated with the degree of initial CD4 T cell depletion during the acute phase of SIV infection [30]. It is possible that the acute phase of retroviral infection is responsible for a shift in the host-pathogen relationship, perhaps via deletion of critical T cell phenotypes that sets up the path to inevitable TB reactivation at some stage [31]. 

In addition to the early impact of HIV seroconversion on immune containment of *M. tuberculosis*, the risk of TB disease continues to rise as CD4 cell counts decrease [32–35]. In an Italian cohort, for example, TB incidence rates among groups of antiretroviral treatment-(ART-) naïve patients with CD4 cell counts of >350, 200–350 and <200 cells/*μ*L were 0.5, 1.8, and 4.7 cases per 100 person-years, respectively [35]. Rates among corresponding groups of patients in South Africa were much higher at 3.6, 12.0, and 17.5 cases/100 person-years, respectively [32], probably reflecting the extremely high rates of infection and ongoing re-exposure to TB in the community [36].

### 4.3. Clinical Observations and Postmortem Studies

It is well established that HIV infection has a profound impact on the spectrum of TB disease, with progressive immunodeficiency being associated with an increased risk of extrapulmonary and disseminated disease [37, 38]. Impairment of the host inflammatory response is associated with much lower rates of cavitation in the lung [39, 40], which in turn accounts for more frequent sputum smear-negative disease and prolonged time to positivity of automated liquid culture of sputum [41, 42]. Despite lower bacillary burden in sputum, however, mycobacterial blood cultures, fine needle aspiration of lymph nodes, and urinary antigen detection provide evidence that the total numbers of mycobacteria in patients with advanced HIV and TB may be very high [38, 42–44].

Postmortem studies of HIV-infected patients conducted in West, East, and Southern Africa also have remarkably consistent findings. Each study found TB to be the commonest cause of death [45–48], being present in over one third of cadavers of HIV-infected patients who died in hospital and in approximately one half of those with AIDS-defining pathology [45–48]. In most cases, disease was disseminated. These data strongly suggest that disseminated HIV-associated TB frequently remains clinically unsuspected and undiagnosed and is a major contributor to HIV-associated mortality in the region. In light of these observations, the WHO estimate of approximately 0.5 million deaths per year in patients with HIV-associated TB [2] may be an underestimate.

### 4.4. “Unmasking” TB during Antiretroviral Treatment

Observational studies of HIV-infected patients initiating ART provide further evidence for the existence of subclinical forms of active TB. In such patients, initiation of ART appears to trigger the presentation of TB during the initial weeks of therapy [49–51]. This phenomenon (often referred to as “unmasking TB”) is thought to be mediated by rapid restoration of *M. tuberculosis*-specific immune responses [52]. In a South African cohort, this phenomenon was estimated to account for approximately 40% of incident TB cases in the first 4 months of ART [53] and this risk was substantially reduced with use of intensive pre-ART TB screening [54]. These data are consistent with the existence of a spectrum of subclinical and clinical TB, the presentation of which may be modified during rapid changes in immune function mediated by ART.

### 4.5. Intensified Case Finding Studies

A meta-analysis of intensified TB case finding studies (*n* = 12) from Africa and Asia found that approximately one fifth of patients with culture-proven HIV-associated TB were free of suggestive symptoms and this proportion was broadly similar across a range of CD4 cell counts [55]. Similarly a systematic review of studies of intensified case finding for HIV-associated TB found that routine microbiological screening without prior patient selection using symptom screening resulted in the identification of additional 4 cases of TB for each 100 patients screened [56]. Thus, prevalence rates of asymptomatic active TB among HIV-infected individuals in high TB burden settings can be considerable [57].

## 5. Implications for Control of HIV-Associated TB

Changing the paradigm of HIV-associated TB into one that encompasses a spectrum of subclinical and symptomatic disease has important potential implications for screening, diagnosis, and prevention using isoniazid preventive therapy and ART.

In the worst affected region, the burden of HIV-associated TB is often far greater than is clinically suspected and this mandates having a low threshold for investigating patients for TB. A major step forward is the development by the World Health Organization and the Centers for Disease Control and Prevention of a high sensitivity symptom screening tool for use in HIV-infected patients [55]. In contrast to the existing strategy of screening for cough of 2-3 weeks duration, this new screening tool identifies any HIV-infected patient with cough (any duration), fever, night sweats, or weight loss, and such patients should be further evaluated for TB disease. Alternatively, in patient groups with the highest risk, routine microbiological screening for TB may be justified [54]. Such approaches should reduce the prevalence of undiagnosed TB, potentially reducing mortality and risk of TB transmission both in health care settings [58] and in the community.

Novel high sensitivity assays are needed for diagnosis of TB and active infection. Much of the developing world remains reliant upon sputum smear microscopy which, in those patient groups with the most advanced immunodeficiency, may have a sensitivity as low as 20% [42, 59]. Expanded availability of culture-based diagnosis is greatly needed, and the development of rapid, simple, and accurate diagnostic tools that can function as point-of-care tests remains an essential goal. In those with most advanced immunodeficiency, development of a point of care assay that detects lipoarabinomannan (LAM) in urine may prove to have some practical utility [42, 43]. However, simplified forms of newer generations of nucleic acid amplification tests have far greater sensitivity and are likely to represent a major step forward [60–62].

New tools are also needed that are able to more precisely define the spectrum of infection and disease states. Such assays might provide prognostic information that indicate the probability of progression of infection to TB disease and also be used to monitor the response of disease to treatment and assess the probability of relapse. With these goals, considerable research efforts are being made to identify predictive biomarkers, which could be either host or pathogen specific [63–65]. Development of such biomarkers could also play a crucial role in expediting the development and evaluation of new vaccines and drugs. 

We have highlighted how disseminated TB frequently remains undiagnosed in patients dying with HIV/AIDS in sub-Saharan Africa. Existing WHO algorithms recommend that seriously ill HIV-infected patients who have cough for 2-3 weeks, are sputum smear-negative, and have not responded to parenteral antibiotics (or treatment of *Pneumocystis jirovecii* pneumonia where appropriate) are recommended to be started on empiric TB treatment [66]. However, in the highest burden settings, a subgroup of HIV-infected patients with advanced immunodeficiency have such a high risk of TB and of associated mortality that they might derive benefit from immediate initiation of empiric TB treatment. Studies exploring such a strategy are needed and one is being planned by the AIDS Clinical Trials Group (ACTG5274 REMEMBER trial) (Mina Hosseinipour, personal communication).

Despite WHO policy recommendations published in 1998 [67], only 0.1% of eligible HIV-infected individuals received IPT in 2007 [68]. One key hindrance to more widespread adoption of this intervention is the difficulty of excluding active TB in HIV-infected individuals with low CD4 cell counts [69]. Since the prevalence of culture-proven active infection and TB disease rises steeply with falling CD4 cell counts, the negative predictive value of screening algorithms is significantly diminished in patient groups with low CD4 cell counts [70]. In patient groups such as those enrolling in ART services in which TB disease prevalence exceeds 10% [56], there is therefore a well-founded reluctance to start such patients on preventive therapy using isoniazid monotherapy despite the lack of objective evidence of an increased risk of generating isoniazid resistant strains of *M. tuberculosis* [71]. Many such patients require ART as the primary intervention anyway and this functions as the key TB preventive intervention [70].

ART is associated with a 67% reduction (range: 54–92%) in TB incidence [70, 72] due to CD4 cell count recovery and restoration of functional anti-mycobacterial immune responses [73–77]. Time-dependent reductions in TB risk [78, 79] are very strongly related to changing absolute CD4 cell counts, with an almost 10-fold difference in adjusted rates comparing patients with counts <100 cells/*μ*L and >500 cells/*μ*L [53]. In those with pulmonary TB disease, ART enhances organism clearance from sputum during TB treatment but the clinical course may also be complicated by immune reconstitution disease [50, 80]. 

In those with active infection, we hypothesize that ART may have two alternative effects. In those with the highest organism load, ART may trigger “unmasking TB” [52]. Alternatively, by enhancing immune-mediated control, ART may cause a shift in the host-pathogen relationship to restore quiescent infection (Figure 1, Table 2). However, during long-term ART, there is no evidence to suggest that complete clearance of infection occurs. Those with optimum CD4 cell count recovery have persistently heightened TB risk that remains several fold higher than background rates [53, 79]. In light of this, concurrent use of IPT during long-term ART would be a logical approach in which complementary immune-mediated and anti-mycobacterial mechanisms were employed to reduce mycobacterial burden [70]. Data from Brazil and Botswana support this suggestion [81, 82].

## 6. Conclusions

The traditional view of binary compartmentalised states of latent TB infection and clinical TB disease is widely acknowledged to be limited and yet this simple paradigm was broadly adequate for designing TB control interventions in the pre-HIV era. However, although these conventional TB control strategies have been associated with reductions in TB rates in much of the world, this is not the case in regions with high HIV prevalence. Here, additional interventions and new tools are desperately required. Understanding “latent TB infection” as a spectrum of immune responses, mycobacterial metabolic activity and organism load redefines approaches to screening, diagnosis and prevention of TB in HIV-infected people. This also redefines the goals for the development of new vaccines and for biomarker discovery and emphasizes the pivotal role of ART in regaining immune control of active infection.

## Figures and Tables

**Table 1:** Evidence that suggests the existing view of “latent tuberculosis infection” and active tuberculosis disease as binary compartmentalised states is oversimplistic.

(1) Risk of developing active tuberculosis in patients with “latent tuberculosis Infection” varies considerably over time, suggesting that “latent” infection is a heterogeneous state
(2) Isoniazid is active against actively replicating organisms and yet reduces TB risk in those with “latent tuberculosis infection”
(3) Isoniazid preventive therapy entails 6–12 months of therapy for good efficacy, possibly suggesting the pool of “latent” mycobacteria cycle through phases of metabolic activity and replication over time
(4) A significant proportion of patients with microbiologically proven pulmonary tuberculosis identified by prevalence surveys have no symptoms
(5) Serological markers are predictive of patients with different stages of tuberculosis infection and disease, including those with asymptomatic active disease
(6) Mycobacterial lesions within tissues from the same individual may represent a wide spectrum, ranging from sterility to multibacillary disease
(7) Discordance between tuberculin skin test and interferon-gamma release assay results cannot simply be explained by differences in specificity. These assays appear to reflect different aspects of immune sensitization which are as yet incompletely understood.

**Table 2:** Proposed framework of potential outcomes of the host-pathogen relationship following exposure to *Mycobacterium tuberculosis* infection. (adapted from [9, 10]).